# Gender Differences in Depression, Coping, Stigma, and Quality of Life in Patients of Vitiligo

**DOI:** 10.1155/2019/6879412

**Published:** 2019-04-02

**Authors:** Neena S. Sawant, Nakul A. Vanjari, Uday Khopkar

**Affiliations:** ^1^Department of Psychiatry, Seth GS Medical College & KEM Hospital, Acharya Dhonde Marg, Parel, Mumbai 400012, India; ^2^Department of Psychiatry, SMBT IMS & RC, Igatpuri, Nashik, India; ^3^Department of Dermatology, Seth GS Medical College & KEM Hospital, Acharya Dhonde Marg, Parel, Mumbai 400012, India

## Abstract

Though vitiligo is one of the psychodermatological disorders which do not cause direct physical impairment, it is cosmetically disfiguring leading to serious psychological problems in daily life. We undertook this research to study patients of vitiligo the prevalence of depression, coping, stigma, and quality of life and comparison of the same in both genders. Patients diagnosed clinically as having vitiligo by consultant dermatologist were enrolled after informed consent and ethics approval. 156 patients were screened, of which 100 satisfying criteria were taken up for the study. A semistructured proforma was designed to collect the necessary information with administration of Beck's depression inventory, participation scale, dermatology life quality index, and adjustment to chronic skin diseases questionnaire. Depression prevalence was 63.64% in females and 42.86% in males (p<0.0457); the total mean BDI scores were significant with females having higher scores than males (p<0.0083). No significant differences were seen on participation scale though 52% females felt stigmatized as compared to 45% males (p <0.5779). While almost 97% of our patients had impaired quality of life there was no significant difference in both genders on the total score (p<0.3547). Females had significantly higher faulty coping style than males with significant differences on all domains and total scores (p< 0.0094). There was a strong association of depression with faulty coping and stigma (p< 0.0001) in both genders. Also association of stigma with quality of life showed highly significant findings in both genders (p< 0.0001) on all the domains of DLQI. This study helps in early identification of psychological problems in vitiligo patients and planning their future course of management, hence improving the prognosis and quality of life.

## 1. Introduction

Vitiligo is an acquired depigmentation disorder affecting 1-4% of the world population with equal distribution in both genders and all ethnic groups [1–3]. In Mexico the prevalence has been reported to be around 4% [4, 5] whereas in India around 8.8% [6] which is the highest incidence of this condition [7]. The depigmentation is caused by functional melanocytes disappearing from the lesional area of the epidermis. Till date no curative treatment is available.

Vitiligo is disfiguring in all races but particularly more so in dark-skinned people because of strong contrast. In India, vitiligo commonly known as leucoderma is unfortunately associated with some religious myths like being a curse of God or a sin for which patients seek help from faith healers and do religious rituals than seeking medical help, thus resulting in social ostracism [8, 9]. Given the visibility of chronic dermatoses, stigmatization becomes a part of daily life in vitiligo patients, which can lead to psychosocial stress and ultimately depression [10–14]. The chronicity, visibility, and relapsing nature of vitiligo impair quality of life and ability to cope [14–20]. Severe depression has been known to lead to suicidal ideations. The reluctance of patients to report their psychological distress is often observed, with consequence of a greater focus on physical symptoms than on psychological aspects like depression, stress, or stigmatization.

Research in vitiligo shows that at least 25% [11] of dermatology patients suffered from significant psychiatric comorbidity and 63% had positive findings on self-reporting questionnaire-24 which was the psychiatric screener [10]. Picardi et al. suggested that untreated comorbid psychiatric disorders may adversely affect the response of the dermatological disorder to prescribed therapies [21]. Treatment of vitiligo patients should address their emotional effects and include tools for psychological intervention, which may ultimately lead to better adaptation to the disease and higher quality of life. Treatment should be aimed at improving the overall quality of life and reducing the stigmatization feeling caused by this chronic disease. The importance of considering the stigmatization experience and coping in vitiligo patients has to be emphasized in both future research and patient treatment.

Though there are Indian studies on several aspects of vitiligo we undertook this research to know about gender differences in the prevalence of depression, stigma, coping, and quality of life in vitiligo patients and to also find the association of depression with coping and stigma as well as the association of stigma with quality of life.

## 2. Methodology

The study was initiated in the dermatology outpatient department of Seth GS Medical College and KEM Hospital, a tertiary general hospital in central Mumbai catering to lower socioeconomic strata of several ethnic groups like Hindus, Muslims, Christians, Jews, Parsis, and Sikhs. The institutional ethics committee of Seth GS Medical College and KEM Hospital gave permission to conduct the study. All patients were diagnosed as having vitiligo by the consultant dermatologist after clinical evaluation based on the presence of a recent depigmented patch in the absence of any contact with substance producing such depigmentation. Wood's lamp accentuation was used as a criterion [22]. Site of lesion was noted as being on areas that were exposed, unexposed, or both exposed and unexposed.

All the patients were explained about the nature of study and its applications and informed consent was obtained from patients who were willing to participate in the study. Patients were initially screened and only those above 18 years of age were enrolled in the study. Data collection was done over a period of 12 months. Those having medical comorbidity like infections, other immunological disorders, or existing psychopathology with ongoing treatment were excluded from the study. 156 patients were screened, of which 100 were enrolled in the study. A proforma was designed to enquire into the sociodemographic details like age, sex, marital status, religion, education, occupation, and income. The socioeconomic strata were assessed using the Kuppuswamy scale [23]. It also included details about vitiligo lesions like age of onset, location and duration, family history of vitiligo, any previous medical or psychiatry illness, and medication history. The clinical variables were compared between male and female patients. Both groups were studied for prevalence of depression, coping, stigma, and quality of life, using the following scales.

### 2.1. Dermatology Life Quality Index (DLQI)

The dermatology life quality index questionnaire is designed for use in adults, i.e., patients over the age of 16. It is self-explanatory and can be simply handed to the patient who is asked to fill it in without the need for detailed explanation. The DLQI is calculated by summing the score of each question resulting in a maximum of 30 and minimum of 0. The DLQI can also be expressed as a percentage of the maximum possible score of 30. Persons who scored ≥10 were considered as DLQI positive cases.

*Meaning of DLQI Scores*

  0-1 = no effect at all on patient's life.
  2-5 = small effect on patient's life.
  6-10 = moderate effect on patient's life.
  11-20 = very large effect on patient's life.
  21-30 = extremely large effect on patient's life.

The higher the score is, the more quality of life is impaired. It is usually completed in one to two minutes. [24]

### 2.2. Participation Scale (PS)

This tool has been validated in India, Nepal, and Brazil. It measures the extent to which people participate in common social events [25]. The key issue of stigma is that it excludes people from participating in such events. The P-scale is an 18-item instrument, which covers eight out of the nine participation domains of the International Classification of Functioning, Disability and Health (ICF) to measure social participation in such stigmatizing diseases. The use of the word participation is based on the ICF terminology and participation restriction is defined as problems an individual may experience with involvement in life situations [26]. A five point rating scale was used to measure the level of participation for each item. For each subject the scores obtained for the 18 items were added up. If the score was 12 or less, people were considered not to have restriction in their domestic and social situation. Scores of 13 to 90 represented restriction at different intensities; 13 to 32 as moderate, 33 to 52 as severe, and 53-90 as extreme restrictions.

### 2.3. Beck's Depression Inventory (BDI)

This scale was devised by Beck in 1961. It contains 21 sentence groups aimed at assessing the level of depression. Observed depression signs are evaluated objectively. The 21 signs of depression included in the scale are sensibility, pessimism, sense of failure, sense of guilt, self-dissatisfaction, self-accusation, desire to commit suicide, hysterical weeping, seizures, nervous breakdown, social retreat, indecisiveness, conflicting self-image, sleep disturbances, tiredness, loss of appetite, loss of weight, psychological complaints, and lack of sexual desire. All the questions were developed based on signs normally seen in depressed individuals. Each category receives a score of 0–3 points. If a subject scores 0–16 points, there is no depression, 17–20 points indicate mild depression, 21-30 points indicate moderate depression, and > 31 points reveal severe depression. Studies using the scale indicate that the BDI is an appropriate method for assessing the signs and levels of depression in a given subject [27].

### 2.4. Adjustment to Chronic Skin Diseases Questionnaire (ASC)

The ASC is a 51-item, fully standardized self-rating instrument used to evaluate coping strategies; the scoring system was also a Likert scale. The ASC consists of six scales: high scores on the “social anxiety/avoidance” scale indicate a frequent avoidance of certain situations due to a fear of rejection; high scores on the “itch-scratch circle” scale a deficient self-control resulting in frequent scratching. High values on the “helplessness” scale correspond to the perception of an almost complete loss of control over the course of the disease; high values on the “anxious-depressive mood” to a problematic adjustment to the skin disorder. High scores on the “impact on quality of life” scale are related to far-reaching objective consequences that influence daily life; high values on the “deficit in active coping” scale to repetitive failing attempts of patients to find an active solution to problems, for example, by researching background information on their skin disease [28].

All analyses were done with SPSS statistical version 17 at 5% significance.

## 3. Results

There were 56 males and 44 females in our study with the male to female ratio being 1.2:1. We found the mean age of male patients of vitiligo to be 35.78 ± 14.23 years and that of females to be 36.88 ± 14.22 years. The age range of all the vitiligo was from 18 to 68 years with majority being in the 18 to 34 years age group for both genders. Majority of the patients in both groups (males 66% and females 64%) were married. 44(78%) males and 37(84%) females were Hindu by religion. Among the minority religion groups there were 9 (16%) Muslims, 2 (3.5%) Sikhs, and 1 Christian in the male group, whereas there were 2 Muslims, 1 Sikh, and 4 Christians in the female group. All males were literate while 34 (77%) females were illiterate. 46 (82%) males were employed while unemployment was seen in 34 (77%) females. About 32 (57%) males and 29 (66%) females had income less than 12000 rupees per month, whereas 14 (25%) males and 13 (30%) females earned between 12000 and 16000 rupees per month (Table 1(a)).

The mean age of onset of vitiligo for males was 28.89 ± 13.54 years and 29.13 ± 13.73 years for females. In both genders we found predominance of vitiligo in the 20 to 40 years of age. The duration of the disease varied from less than one year to several years. Majority of our patients (47 (84%) males and 40 (91%) females) had illness duration of more than 1 year indicating the chronic nature of the illness. Family history of vitiligo was found in 13 (23%) males and 11 (25%) females. Most of our patients (52% male and 68% female) had lesions on both exposed and nonexposed parts of the body (Table 1(b)).

When all the patients were assessed for prevalence of depression using BDI, 52% of the total 100 patients were found to be depressed. The gender differences for depression revealed a higher prevalence of 28(63.64%) in females as compared to 24 (42.86%) in males which was statistically significant. When BDI total scores were compared for gender differences a highly statistical difference was seen with the female patients having a mean BDI total score of 28.04 ± 20.5 as compared to males who had a mean score 17.3 ± 17.1. On assessing for severity of depression as per BDI in both groups, about 23 (82%) of females had severe to extreme depression as compared to 14 (58%) males. 10 (42%) males were having predominantly borderline to moderate depression as compared to 5 (18%) females indicating again that the severity of the depression was more in females than males (Table 2).

When we assessed our patients for prevalence of stigma, 48% of the total 100 patients were experiencing it in the form of participation restriction. On assessing for gender differences, 23 (52%) of females as compared to 25 (45%) males reported restriction in activities as per PS due to the stigma faced. (Table 3)

When both groups were compared for differences in coping then, a highly significant difference (U score-857 and p value-0.0094) was seen between the genders with the females having a higher score indicating faulty coping as per the ASC. Further assessing gender differences on the various subdomains of ASC revealed significant differences with a female preponderance on social anxiety (U score-879.5 and p value-0.0145), helplessness (U score-892 and p value-0.0183), anxious-depressive mood (U score-909 and p value-0.0247), and impact on quality of life (U score-857.5 and p value-0.066) except for itch-scratch which was not statistically significant (Table 4).

We found almost all our patients (males 96.43% and females 97.73%) to be having impaired quality of life when assessed using dermatology life quality index. When both groups were assessed for differences in the domain scores of the DLQI, then no significant differences were seen on the various domains, namely, feeling and symptoms (U score-1076 and p value-0.2708), daily activities (U score-1009.5 and p value-0.1144), leisure ( U score-1191 and p value-0.7710), work and school (U score-1213 and p value-0.8889), personal relationship ( U score-1229 and p value-0.9885), treatment ( U score-1029 and p value-0.1304), and also the total score (U score-1098 and p value-0.3547). The total DLQI scores did not show any statistically significant differences among males and females indicating that the score was not influenced by gender.

Among patients with impaired quality of life, no statistically significant differences were noted between the two genders. Majority were having very large to extremely large impairment in their quality of life with 48% males and 53% females falling under these two categories indicating the large impact of vitiligo on quality of life. The highest individual mean score was obtained on the treatment question while the lowest was on sport activity question indicating most and least impairment in the above respective areas (Table 5).

When both groups were assessed for association of depression with coping, then highly significant findings were seen in both genders on all the domains of ASC (Table 6).

Likewise association of depression with stigma showed highly significant findings for both genders (Table 7).

Also assessment for association of stigma with quality of life showed highly significant findings in both genders on all the domains of DLQI (Table 8).

## 4. Discussion

Researchers have found mean age of patients with vitiligo to be ranging from 29.72 ± 7.01 years [14], and 43.8±12.48 years [10] which are in keeping with our findings. However no gender studies showing an earlier onset in males or females are available. Several researchers have reported male to female ratio similar to our findings. Pichaimuthu et al. in his sample of 55% males and 45% females also had a male to female ratio of 1.2:1 [29]. Sangma et al. [14] reported a male to female ratio of 1.4:1, though female predominance has been reported in some studies which could be due to the women's tendency to give greater attention to cosmetic defects as compared to men [13].

Marriage rate was seen to be 69% [30] in vitiligo patients in previous studies which is in keeping with our findings whereas Mishra et al. [20] reported 48% to be married. The high marriage percentage could be explained by the cultural background, as in India, marriages occur in the early ages from 18 to 25 years. Our religion percentage was in keeping with cultural diversity of India which showed almost 81% Hindus followed by minority groups, namely, Muslims, Sikh, and Christians. Majority of our patients were from the upper lower and lower middle socioeconomic strata having income mostly less than Rs. 12000 or up to Rs.16000 per month, reflecting the population attending a tertiary care general hospital, where medical services and medicines are supplied free of cost to the general public. Also in our study, more male patients were educated and employed than females. Other studies have found vitiligo predominantly in lower and middle classes (72 %) with a higher prevalence in the upper classes (28%) as compared to our study [31].

Pichaimuthu et al. [29] found 35% of patients having illness duration less than 1 year, whereas we found the same in only 13% of the patients. Vitiligo has a polygenic or autosomal dominant inheritance pattern with incomplete penetration and variable expression. Our finding about family history is in keeping with those reported by Kruger and Schallreuter [11]. Studies have also shown a relatively early onset of vitiligo symptoms in patients having family history of vitiligo [32]; however it was not reflected in our study. Positive family history is considered to be poor prognostic factor for vitiligo.

The site of lesion largely varies in different studies. Our findings are different from other researchers who found 57% patients to be having lesions on exposed parts like face, neck, nape of the neck, forearms, hands, fingers tips, foot, toes, and 39% patients having lesions on both exposed and nonexposed body parts [29]. Kruger and Schallreuter in their study reported most common sites as head (88.5%), hands (83.3%), arms (76.0%), legs (75.0%) trunk (70.8%), and neck (57.3%) [11]. The probable reason for our findings of higher prevalence in both exposed and nonexposed body parts could be the chronic nature of illness with progression.

Though vitiligo is one of the psychodermatological disorders which do not cause direct physical impairment, it is cosmetically disfiguring leading to serious psychological problems in daily life [3, 10, 11]. Various psychological effects of vitiligo include low self-esteem, social anxiety, isolation, depression, impaired quality of life, etc. The prevalence of psychiatric morbidity associated with vitiligo ranged from 56% to 79% in India [12]. Several meta-analyses have shown that prevalence of clinical depression as per standard criteria was 8% which increased to 33% on using scales [22, 33]; on diagnostic codes the pooled prevalence of depression among patients with vitiligo was 0·253 [95% confidence interval (CI) 0·16–0·34;* P* < 0·001)] while with self‐reported questionnaires, the pooled prevalence of depressive symptoms was 0·336 (95% CI 0·25–0·42;* P* < 0·001) [34, 35]. Similarly Osinubi et al. reported the pooled prevalence using depression-specific and anxiety-specific questionnaires as 0·29 [95% confidence interval (CI) 0·21-0·38] and 0·33 (95% CI 0·18-0·49), respectively [36]. Several researchers have reported depression in vitiligo patients ranging from 18-37% [37], 62.2% [11], to 79% [14] which is similar to our findings. Depression could have a cause or effect with vitiligo as studied by many researchers [2, 12, 13, 25]. BDI total scores indicated that females were affected more significantly and severely than males. Generally females experience more intense depressive features because of the more stress experienced and have a greater reactivity to it with a higher rate of body dissatisfaction and low self-esteem [11, 33]. Of the total patients who were depressed about 71% had severe to extreme depression, 21% had moderate depression, and 8% had borderline depression. However we did not analyze the association between severity of vitiligo and depression.

Vitiligo is known to be associated with stigma. Other researchers from India have reported a lower stigma prevalence of 17% as compared to our findings in the vitiligo patients [29]. However, Kent had found a higher prevalence of stigma in 63% of his patients [38]. In our study participation restriction was experienced in areas like social interaction, work opportunities, religious activity, going out in public places, meeting new people, etc. by all the patients. Kruger and Schallreuter reported that 90% of patients experienced being asked questions by strangers for their white spots and 50-60% experienced rudeness and staring looks due to which they had avoidance and concealing behaviours [11]. This could be one of the reasons why majority of our patients experienced stigma, though we did not get any statistically significant difference in both genders. We did not study for the association between severity of vitiligo and stigmatization.

On ASC scale, females experienced significant social anxiety and avoidance as compared to males probably due to greater cosmetic awareness with avoidance due to feeling of looking unattractive or being stared by others. This resulted in making them avoid meeting new people, withdrawing from family, being sexually inhibited, etc. As compared to other skin disorders there was no irresistible itching or scratching seen in vitiligo patients and hence it was not a significant finding in our study. However in a study by Leibovici et al. on comparing for coping differences in psoriasis and atopic dermatitis a significant difference was seen with psoriatic patients having more social avoidance and greater impact on quality of life on the domains of ASC than the atopic dermatitis patients [39]. Rahman et al. also found itching in only 16% of patients with vitiligo [31]. On the domain of helplessness patients experienced ruminations, felt desperate, worried about illness, and future with a lot of attention and time spent on inspecting their skin. Females outscored males significantly in helplessness scores, indicating higher severity of symptoms in them with an almost complete loss of control over the course of the disease again. Our finding is in keeping with Schmid-Ott et al. who also felt that the female's retreat and low composure due to the stigmatization experience lead to more perceived helplessness in coping with the disease [40]. Anxious-depressive mood domain of ASC scale showed that patients who experienced nervousness, tiredness, and lack of concentration got irritated and upset easily. Females were significantly more depressed and anxious than males. Higher scores among females were also reported on anxious-depressive mood domain suggesting negative self-evaluation and problematic adjustment to the skin disorder [40]. On the domain of impact on the quality of life of ASC scale, patients felt that chronic illnesses were expensive; they could not do certain jobs and had personal and work related difficulties. We found females having significantly higher scores than males on the impact on quality of life domain.

The chronic, unpredictable nature of the disease and the lack of a universally effective treatment are disempowering for patients with vitiligo and leads to impaired quality of life [30]. Our finding is in keeping with that of Talsania et al. who found impaired quality of life in 96% of their vitiligo patients [41]. Our findings about gender differences in domains of DLQI are similar to that of Parsad D et al. [17] and Karelson et al. [32]. Parsad et al. in their study on Indian vitiligo patients found higher mean total DLQI scores (10.67 ± 4.56) which was associated with darker skin as compared to fairer skin [17]. They postulated that the dark-skinned people attracted more unwanted attention which was emotionally disturbing and upsetting. Mishra et al. [20] reported a lower mean DLQI score of 6.8 in their patients.

On the domain of symptoms and feelings of DLQI, patients felt self-conscious and embarrassed about the disease and some had itching and pain over the lesions. The females scored more than the males probably due to cosmetic and aesthetic orientation as expected. Similar findings were reported by Hedayat et al. [42]. On the domain of daily activities of DLQI, patients had difficulties at looking after homework, going out for shopping, and their clothing style was also affected by lesions, as many of them tried to hide the lesions by wearing full clothes. Leisure domain of DLQI indicated that the patients had many times difficulties in their social and leisure activities and some of them were not able to play or participate in sport activities because of the vitiligo. Work and school domain of DLQI showed that some of the patients experienced problems at work and school as they were not able to concentrate enough and had difficulties in completing their task. Males had higher mean scores than females. On the domain of personal relationship, many faced problems in keeping touch with close friends or relatives. Also some claimed to have difficulties in sexual relationship as they felt embarrassed and less enthusiastic due to the lesions. On this domain, the males in our study group scored more than females and this was also reported by Porter et al. who observed more frequent embarrassment in sexual relationships among men with vitiligo [43]. Vernwal reported that vitiligo affected marital, sex life, and intimacy and disrupted the social relationship and created a vicious stress-vitiligo cycle [37]. Majority of the patients had to spend lot of time and money for the treatment as long follow-ups were needed due to chronic nature of illness. Also, their daily routine and work were disturbed due to repeated hospital visits. Females in our groups scored more than males as they expressed difficulty in leaving household chores for follow up visits.

Our results indicate that depressed patients were having significantly faulty coping styles or vice versa. Picardi et al. found increased psychiatric morbidity in female outpatients with skin lesions and reported that alexithymia, insecure attachment, and poor social support appeared to increase susceptibility to vitiligo, due to reduced ability to cope effectively with stress [44, 45]. Gieler et al. suggested that an early improvement in coping strategies by using psychotherapeutic/psychosomatic measures could help in reducing higher scores in anxious and depressed vitiligo patients [18]. Higher scores on the ‘anxious-depressive mood' scale and the ‘helplessness' scale of the ASC imply a strongly negative self-evaluation of affected persons resulting in retreat and avoidance and reduced quality of life which was significantly seen in both our groups and reported by other researchers [40, 45].

All those who were depressed experienced more stigma and showed restrictions in job or work opportunities, visiting markets or bazaar, schools, shops, offices, new people, participating in festive and rituals, chatting, or meeting friends or neighbours. Also many claimed that they had less respect in community as compared to others and had difficulty in maintaining long-term relationship with their partners. All stigmatized patients in our study were having significantly impaired quality of life or vice versa in both genders. Studies have shown that stigmatized and embarrassed patients experience low self-esteem and poor quality of life which lead to significantly higher depression rates among them [46].

Overall women's greater reactivity compared to men has been attributed to gender differences in biological and emotional responses, self-concepts, and coping styles which could be one of the reasons why the females in our sample experienced more depression, poor coping, and quality of life, with a chronic illness like vitiligo probably exacerbating it [47, 48].

## 5. Conclusions

This study helps to understand the impact of vitiligo and gender based differences in quality of life, coping, psychiatric comorbidities like depression and stigma faced. The results of study clearly support the notion that treatment of vitiligo patients should address the emotional effects and include tools for psychological intervention, which may ultimately lead to better adaptation to the disease and coping, thus improving the patients overall quality of life. Liaison with the psychiatrist is important for early assessment of depressive symptoms and considering both psychotherapeutic and psychopharmacological treatment options. Long-term prospective studies in different chronic skin conditions would help in the better understanding of the gender based differences.

## Figures and Tables

**Table:** (a) Demographic variables

Variables		Male	Female
		(n=56)	(n=44 )
Sex		56	44

Age	Mean	35.786	36.886
	SD	14.238	14.226

Age range	18-34 years	31 (55.36%)	20 (45.46%)
	35-51 years	15 (26.78%)	16 (36.36%)
	52-68 years	10 (17.86%)	8 (18.18%)

Marital status	Married	37 (66.08 %)	28 (63.64 %)
	Unmarried	19 (33.92 %)	16 (36.36 %)

Religion	Hindu	44 (78.57 %)	37 (84.09 %)
	Others	12 (21.43%)	7 (15.91%)

Education	Literate	56 (100%)	10 (22.72%)
	Illiterate	0 (0%)	34 (77.28%)

Occupation	Employed	46 (82.14%)	10 (22.72%)
	Unemployed	10 (17.86%)	34 (77.28%)

Income in rupees per month	< 12000 Rs	32 (57.14%)	29 (65.91%)
	12000-16000 Rs	14 (25%)	13 (29.54%)
	>16000 Rs	10 (17.86%)	2 (4.55%)

**Table:** (b) Illness variables

Variable		Males	Females
		(n= 56)	(n= 44)
Age of onset of vitiligo	Mean	28.89	29.13
	SD	13.54	13.73

Age range of onset of vitiligo	0-20 years	20 (35.71%)	14 (31.82%)
	21-40 years	24 (42.86%)	20 (45.45%)
	41-60 years	12 (21.43%)	10 (22.73%)

Duration of vitiligo	< 1 year	9 (16.07%)	4 (9.09%)
	> 1 year	47 (83.93%)	40 (90.91%)

Family history of vitiligo	Present	13 (23.21 %)	11 (25 %)
	Absent	43 (76.79 %)	33 (75 %)

Site of lesion of vitiligo	Exposed	21 (37.5%)	8 (18.18%)
	Unexposed	6 (10.71%)	6 (13.64%)
	Exposed + Unexposed	29 (51.79%)	30 (68.18%)

**Table 2:** Prevalence and severity of depression as per BDI.

*Depression as per BDI*	*Males [n= 56 (*%*)]*		*Females [n= 44 (*%*)]*		*p value*
	*Present*	*Absent*	*Present *	*Absent*	0.0457*∗*( Fisher's test)
	24 (42.86%)	32 (57.14%)	28 (63.64%)	16 (36.36%)	

*Severity of Depression*	*Males [n= 24 (*%*)]*		*Females [n= 28(*%*)]*		0.1375 (Chi square for independence)
Borderline depression	2 (8.33%)		2 (7.15%)		
Moderate depression	8 (33.33%)		3 (10.71%)		
Severe depression	7 (29.17%)		7 (25%)		
Extreme depression	7 (29.17%)		16 (57.14%)		

*BDI Total scores*	*Mean ± SD*		*Mean ± SD*		Mann WhitneyU- 852.00, 0.0083*∗*
	17.375± 17.168		28.045±20.505		

**Table 3:** Prevalence and severity of stigma as per participation scale.

*Stigma as per PS*	*Males[ n= 56 (*%*)]*		*Females [n= 44 (*%*)]*		*p value*
	*Present*	*Absent*	*Present *	*Absent*	0.5779 ( Fisher's test)
	25 (44.64%)	31 (55.36%)	23 (52.27%)	21 (47.73%)	

*Restriction severity *	*Males [n= 25 (*%*)]*		*Females [n= 23 (*%*)] *		
Mild Restriction	7 (28%)		2 (8.69%)		
Moderate Restriction	10 (40%)		12 (52.17%)		
Severe Restriction	8 (32%)		8 (34.78%)		
Extreme Restriction	0 (0%)		(4.34%)		

*PS Total scores*	*Mean ± SD*		*Mean ± SD*		Mann WhitneyU-1008.00, 0.1183
	14.054 ± 14.444		18.182 ± 15.358		

**Table 4:** Gender differences for coping as per ASC.

Domain	Males	Females	Mann-Whitney U score, p value
	n=56	n=44	
	Mean ± SD	Mean± SD	
Social anxiety/ avoidance	33.518± 14.025	40.591± 15.083	879.50
			0.0145*∗*

Itch-Scratch	11.875± 5.663	13.773± 6.626	1024.50
			0.1045

Helplessness	22.464± 9.796	27.432± 10.643	892.00
			0.0183*∗*

Anxious-Depressive mood	16.036± 7.913	20.205± 8.938	909.00
			0.0247*∗*

Impact on Quality of life	11.232± 4.191	13.841± 4.861	842.00
			0.066*∗*

Total Score	95.125± 38.902	115.84± 43.253	857.50
			0.0094*∗*

**Table 5:** Prevalence and severity of impairment in quality of life (QOL) as per DLQI.

*Impairment in QoL*	*Males [n= 56 (*%*)]*		*Females [ n= 44 (*%*)]*		*p value*
	*Present*	*Absent*	*Present*	*Absent*	1.00 ( Fisher's test)
	54 (96.43%)	2 (3.57%)	43 (97.73%)	1 (2.27%)	

*DLQI Domain scores*	*Mean ± SD*		*Mean ± SD*		

Feelings & symptoms	2.339± 1.431		2.682±1.475		Mann Whitney U-1076.0, 0.2708

Daily activities	2.304±2.288		3.091±2.341		Mann Whitney U1009.5, 0.1144

Leisure	1.643±1.752		1.614±1.385		Mann Whitney U-1191.0, 0.7710

Work & School	0.8036±0.9802		0.7955±0.9042		Mann Whitney U- 1213.0, 0.8889

Personal relationships	1.607±1.670		1.591±1.661		Mann Whitney U- 1229.5, 0.9885

Treatment	2.036±0.8304		2.295±0.7015		Mann Whitney U- 1029.0, 0.1304

*Total DLQI Score*	10.714±7.827		11.977±7.605		Mann Whitney U- 1098.5, 0.3547

*Severity of Impairment*	*Males [n= 56 (*%*)]*		*Females [n= 44 (*%*)]*		0.8257 (Chi square test for independence)
Small impairment	19 (35.18%)		15 (34.88%)		
Moderate impairment	9 (16.67%)		5 (11.63%)		
Very Large impairment	20 (37.04%)		16 (37.21%)		
Extremely Large impairment	6 (11.11%)		7(16.28%)		

**Table 6:** Association of depression with coping in both genders.

Variable		Males n= 56		Females n= 44	
		Mean± SD	Spearman r, p value	Mean± SD	Spearman r, p value
BDI total		17.375 ± 17.168		28.045± 20.505	

ASC domains	Social anxiety	33.518±14.025	0.9318, < 0.0001*∗*	40.591±15.083	0.9123 < 0.0001*∗*
	Itch- scratch	11.875±5.663	0.5727 < 0.0001*∗*	13.773±6.626	0.6818 < 0.0001*∗*
	Helplessness	22.464±9.796	0.8880 < 0.0001*∗*	27.432±10.643	0.9510 < 0.0001*∗*
	Anxious- depressive mood	16.036±7.913	0.8923 < 0.0001*∗*	20.205±8.938	0.9448 < 0.0001*∗*
	Impact on quality of life	11.232±4.191	0.8935 < 0.0001*∗*	13.841±4.861	0.9400 < 0.0001*∗*
	ASC total	95.125±38.902	0.9187 < 0.0001*∗*	115.84±43.253	0.9460 < 0.0001*∗*

**Table 7:** Association of depression with stigma in both genders.

Variable	Males n= 56		Females n= 44	
	Mean± SD	Spearman r, p value	Mean± SD	Spearman r, p value
BDI total	17.375 ± 17.168	0.8542, < 0.0001*∗*	28.045±20.505	0.8961 < 0.0001*∗*
PS total	14.054 ± 14.444		18.182±15.358	

**Table 8:** Association of stigma with quality of life in both genders.

Variable		Males n= 56		Females n= 44	
		Mean±SD	Spearman r, p value	Mean±SD	Spearman r, p value
PS total		14.054 ±14.444		18.182± 15.358	

DLQI Domains	Feelings & symptoms	2.339±1.431	0.7312 <0.0001	2.682±1.475	0.7936 <0.0001
	Daily activities	2.304±2.288	0.8181 <0.0001	3.091±2.341	0.8941 <0.0001
	Leisure	1.643±1.752	0.8084 <0.0001	1.614±1.385	0.8934 <0.0001
	Work & School	0.8036 ±0.9802	0.6930 <0.0001	0.7955±0.9042	0.6795 <0.0001
	Personal relationships	1.607±1.670	0.7199 <0.0001	1.591±1.661	0.8247 <0.0001
	Treatment	2.036±0.8304	0.6528 <0.0001	2.295±0.7015	0.4765 <0.0011
	Total score	10.714±7.827	0.8111 <0.0001	11.977±7.605	0.8705 <0.0001

## Data Availability

The data used to support the findings of this study are included within the article.

## Additional Points

*Limitations. *(1) The sample size was small and the cases belonged to a tertiary care centre which did not reflect the prevalence in the general population. (2) The aims of the study were not analyzed with respect to activity (i.e., active or stable), severity, and type (segmental or nonsegmental) of vitiligo which would improve our understanding of the impact of vitiligo. (3) Study population included only adults and hence could not establish findings in children and adolescent population.

## References

[1] Grimes P. E. (2016). *Vitiligo: Pathogenesis, clinical features, and diagnosis*.

[2] Grimes P. E., Miller M. M. (2018). Vitiligo: Patient stories, self-esteem, and the psychological burden of disease. *International Journal of Women's Dermatology*.

[3] Ezzedine K., Grimes P. E., Meurant J.-M. (2015). Living with vitiligo: Results from a national survey indicate differences between skin phototypes. *British Journal of Dermatology*.

[4] Canizares O. (1960). Geographic dermatology: Mexico and central america: The influence of geographic factors on skin diseases. *JAMA Dermatology*.

[5] Salinas-Santander M., Sánchez-Domínguez C., Cantú-Salinas C. (2014). Vitíligo: factores asociados con su aparición en pacientes del noreste de México. *Dermatología Revista Mexicana*.

[6] Shajil E. M., Chatterjee S., Agrawal D., Bagchi T., Begum R. (2006). Vitiligo: pathomechanisms and genetic polymorphism of susceptible genes. *Indian Journal of Experimental Biology (IJEB)*.

[7] Dhar S., Dutta P., Malakar R., Valia R. G., Valia A. R. (2008). Pigmentary disorders. *in IADVL Textbook of Dermatology*.

[8] Abraham S., Raghavan P. (2015). Myths and facts about vitiligo: An epidemiological study. *Indian Journal of Pharmaceutical Sciences*.

[9] Eram U. (2017). Review Article on Beliefs and Myths of Vitiligo. *International Journal of Engineering Technology Science and Research*.

[10] Sarkar S., Sarkar T., Sarkar A., Das S. (2018). Vitiligo and psychiatric morbidity: A profile from a vitiligo clinic of a rural-based tertiary care center of eastern India. *Indian Journal of Dermatology*.

[11] Krüger C., Schallreuter K. (2015). Stigmatisation, avoidance behaviour and difficulties in coping are common among adult patients with vitiligo. *Acta Dermato-Venereologica*.

[12] Mattoo S. K., Handa S., Kaur I., Gupta N., Malhotra R. (2002). Psychiatric morbidity in vitiligo: Prevalence and correlates in India. *Journal of the European Academy of Dermatology and Venereology*.

[13] Tripathi K. M., Arya S., Singh V. (2018). Frequency of occurrence of different types of leucoderma and vitiligo, rishi, dasna, ghaziabad. *International Journal of Current Microbiology and Applied Sciences*.

[14] Sangma L. N., Nath J., Bhagabati D. (2015). Quality of life and psychological morbidity in vitiligo patients: A study in a teaching hospital from north-east India. *Indian Journal of Dermatology*.

[15] Thompson A. R., Clarke S. A., Newell R. J., Gawkrodger D. J. (2010). Vitiligo linked to stigmatization in British South Asian women: A qualitative study of the experiences of living with vitiligo. *British Journal of Dermatology*.

[16] Bae J. M., Lee S. C., Kim T. H., Yeom S. D., Shin J. H., Lee W. J. (2018). Factors affecting the quality of life in patients with vitiligo: a nationwide study. *British Journalof Dermatology*.

[17] Parsad D., Pandhi R., Dogra S., Kanwar A. J., Kumar B. (2003). Dermatology life quality index score in vitiligo and its impact on the treatment outcome. *British Journal of Dermatology*.

[18] Gieler U., Brosig B., Schneider U. (2000). Vitiligo-coping behavior. *Dermatology and Psychosomatics*.

[19] Ongenae K., Van Geel N., De Schepper S., Naeyaert J.-M. (2005). Effect of vitiligo on self-reported health-related quality of life. *British Journal of Dermatology*.

[20] Mishra N., Rastogi M. K., Gahalaut P., Agrawal S. (2014). Dermatology specific quality of life in vitiligo patients and its relation with various variables: A hospital based crosssectional study. *Journal of Clinical and Diagnostic Research*.

[21] Picardi A., Abeni D., Renzi C., Braga M., Melchi C. F., Pasquini P. (2003). Treatment outcome and incidence of psychiatric disorders in dermatological out-patients. *Journal of the European Academy of Dermatology and Venereology*.

[22] Gawkrodger D. J., Ormerod A. D., Shaw L. (2008). Guideline for the diagnosis and management of vitiligo. *British Journal of Dermatology*.

[23] Dudala S. (2013). Updated Kuppuswamy′s socioeconomic scale—A revision of economic parameter for 2012. *Journal of Dr. NTR University of Health Sciences*.

[24] Finlay A. Y., Khan G. K. (1994). Dermatology Life Quality Index (DLQI)—a simple practical measure for routine clinical use. *Clinical and Experimental Dermatology*.

[25] Van Brakel W. H., Anderson A. M., Mutatkar R. K. (2006). The participation scale: Measuring a key concept in public health. *Disability and Rehabilitation*.

[26] World Health Organisation (2001). International classification of functioning, disability and health - short version.

[27] Beck A. T., Steer R. A., Brown G. K. (1996). *BDI–II, BeckDepression Inventory: Manual, Harcourt Brace*.

[28] Stangier U., Ehlers A., Gieler U. (2003). Measuring adjustment to chronic skin disorders: validation of a self-report measure. *Psychological Assessment*.

[29] Pichaimuthu R., Ramaswamy P., Bikash K., Joseph R. (2011). A measurement of the stigma among vitiligo and psoriasis patients in India. *Indian Journal of Dermatology, Venereology and Leprology*.

[30] Balaban Ö. D., Atagün M. İ., Özgüven H. D., Özsan H. H. (2011). Psychiatric morbidity in patients with vitiligo / Vitiligolu hastalarda psikiyatrik morbidite. *Dusunen Adam: The Journal of Psychiatry and Neurological Sciences*.

[31] Rahman M., Amin M., Rahman M., Satter M. (2013). A demographic study on vitiligo sheti in Bangladesh. *International Journal of Research in Medical Sciences*.

[32] Karelson M., Silm H., Salum T., Kõks S., Kingo K. (2012). Differences between familial and sporadic cases of vitiligo. *Journal of the European Academy of Dermatology and Venereology*.

[33] Wang G., Qiu D., Yang H., Liu W. (2018). The prevalence and odds of depression in patients with vitiligo: a meta-analysis. *Journal of the European Academy of Dermatology and Venereology*.

[34] Lai Y. C., Yew Y. W., Kennedy C., Schwartz R. A. (2017). Vitiligo and depression: a systematic review and meta-analysis of observational studies. *British Journal of Dermatology*.

[35] Sharma V. K., Bhatia R. (2017). Vitiligo and the psyche. *British Journal of Dermatology*.

[36] Osinubi O., Grainge M. J., Hong L. (2018). The prevalence of psychological comorbidity in people with vitiligo: a systematic review and meta-analysis. *British Journal of Dermatology*.

[37] Vernwal D. (2017). A study of anxiety and depression in Vitiligo patients: New challenges to treat. *European Psychiatry*.

[38] Kent G. (1999). Correlates of perceived stigma in vitiligo. *Psychology & Health*.

[39] Leibovici V., Canetti L., Yahalomi S. (2010). Well being, psychopathology and coping strategies in psoriasis compared with atopic dermatitis: A controlled study. *Journal of the European Academy of Dermatology and Venereology*.

[40] Schmid-Ott G., Künsebeck H. W., Jecht E. (2007). Stigmatization experience, coping and sense of coherence in vitiligo patients. *Journal of the EuropeanAcademy of Dermatology & Venereology*.

[41] Talsania N., Lamb B., Bewley A. (2010). Vitiligo is more than skin deep: A survey of members of the Vitiligo Society. *Clinical and Experimental Dermatology*.

[42] Hedayat K., Karbakhsh M., Ghiasi M. (2016). Quality of life in patients with vitiligo: A cross-sectional study based on Vitiligo Quality of Life index (VitiQoL). *Health and Quality of Life Outcomes*.

[43] Porter J. R., Beuf A. H., Lerner A. B., Nordlund J. J. (1990). The effect of vitiligo on sexual relationships. *Journal of the American Academy of Dermatology*.

[44] Picardi A., Abeni D., Renzi C., Braga M., Puddu P., Pasquini P. (2001). Increased psychiatric morbidity in female outpatients with skin lesions on visible parts of the body. *Acta Dermato-Venereologica*.

[45] Picardi A., Pasquini P., Cattaruzza M. S. (2003). Stressful life events, social support, attachment security and alexithymia in vitiligo: A case-control study. *Psychotherapy and Psychosomatics*.

[46] Kim D. Y., Lee J. W., Whang S. H., Park Y. K., Hann S., Shin Y. J. (2009). Quality of life for Korean patients with vitiligo: Skindex-29 and its correlation with clinical profiles. *The Journal of Dermatology*.

[47] Deng Y., Chang L., Yang M., Huo M., Zhou R., Eder A. B. (2016). Gender differences in emotional response: inconsistency between experience and expressivity. *PLoS ONE*.

[48] Bianchin M., Angrilli A. (2011). Gender differences in emotional responses: Apsychophysiological study. *Physiology &amp; Behavior*.

